# Comprehensive Per- and Polyfluorinated Substances Profiling in Beverages: Simultaneous Quantification of Ultrashort-Chain to Long-Chain Compounds in Ready-to-Drink Teas and Fruit Juices

**DOI:** 10.3390/toxics14050422

**Published:** 2026-05-12

**Authors:** Shun-Hsin Liang, Justin A. Steimling

**Affiliations:** Restek Corporation, 110 Benner Circle, Bellefonte, PA 16823, USA; justin.steimling@restek.com

**Keywords:** per- and polyfluoroalkyl substances (PFAS), ultrashort-chain, beverage, tea, juice

## Abstract

Ultrashort-chain (USC) per- and polyfluoroalkyl substances (PFAS) are highly polar, mobile, and persistent emerging pollutants. While the environmental distribution of USC species is well-documented, their presence in widely consumed beverages remains under-characterized due to the analytical difficulty of capturing such highly polar species. This study established a robust workflow for the simultaneous determination of C1 to C14 perfluoroalkyl carboxylic and sulfonic acids, alongside other PFAS classes, in diverse beverage matrices including teas and fruit juices. Chromatographic separation was achieved using a mixed-mode inert-coated alkyl-phase LC column to enhance USC retention while maintaining performance for longer-chain analytes. A high-throughput, minimal-handling sample preparation was optimized to mitigate matrix effects and contamination. Method performance was evaluated using fortified beverage samples across 2–500 ng/L, with calibration ranges of 1–2000 ng/L and incorporation of 13 isotopically labeled internal standards. Results demonstrated acceptable accuracy (recoveries within 30% of nominal values) and optimal precision (%RSD < 12%). Application to commercial samples revealed frequent PFAS occurrence, specifically highlighting the prevalence of previously overlooked USC species in the human diet. These results demonstrate that ready-to-drink beverages are a significant pathway for human exposure, necessitating the inclusion of USC compounds in future food safety monitoring and risk assessments.

## 1. Introduction

Per- and polyfluoroalkyl substances (PFAS) represent a large family of synthetic chemicals used extensively in industry and consumer products for their chemical stability and resistance to water, oil, and heat [1]. Their widespread production and persistence have resulted in global dissemination in soil, water bodies, and the food supply, raising growing concerns about chronic dietary and environmental exposures [2,3,4,5]. While many PFAS studies have historically focused on short- and long-chain compounds, emerging evidence indicates that ultrashort-chain (USC) PFAS, defined as those containing one to three perfluorinated carbon atoms, may contribute substantially to overall contamination because of their exceptional mobility, environmental persistence, and increasing relevance to dietary exposure [6]. These C1–C3 compounds comprise trifluoroacetic acid (TFA), perfluoropropanoic acid (PFPrA), trifluoromethanesulfonic acid (TFMS), perfluoroethanesulfonic acid (PFEtS), and perfluoropropanesulfonic acid (PFPrS), which are increasingly recognized as major contributors to overall PFAS burdens in aquatic environments [7,8,9]. Their formation pathways are diverse and often linked to atmospheric chemistry, industrial use, and degradation of fluorinated precursors. TFA is the most extensively documented USC compound, formed predominantly through the atmospheric oxidation of several hydrofluorocarbons (HFCs) and hydrochlorofluorocarbons (HCFCs) used as refrigerants [10,11]. TFA can also form through the degradation of several anthropogenic chemicals, including specific pharmaceuticals, pesticides, and fluorinated polymers [12]. Its exceptional environmental stability enables long-term persistence in soil, surface water, groundwater, and even the atmosphere. PFPrA shares similar origins, forming through the atmospheric breakdown of select HFCs and HCFCs [13], as well as through the degradation of fluorinated compounds incorporated into firefighting fluids [14]. It is frequently reported as the dominant PFAS in precipitation, accounting for up to 45% of total PFAS measured in rain and snow samples [9]. TFMS, a strong sulfonic acid used as a catalyst in the manufacture of pharmaceuticals and electrochemical materials [15], can enter the environment through releases from these processes. It has been detected near firefighting training facilities and hazardous waste operations [16], indicating additional sources linked to thermal or oxidative breakdown of fluorinated materials. PFEtS and PFPrS have been identified as components of aqueous film-forming foams and detected in groundwater surrounding U.S. military installations [17]. They can also be generated as byproducts of electrochemical fluorination during the production of various fluorinated surfactants [18]. Collectively, these pathways contribute to rising levels of USC PFAS in aquatic systems. A recent study has documented the widespread occurrence of TFA, PFPrA, and TFMS in household tap water [19], highlighting their environmental persistence and growing relevance to human exposure.

Current analytical approaches for PFAS determination in foods often exclude USC compounds from the target lists, hindering a complete assessment of their contribution to overall dietary exposure [20]. A broad range of beverage products—such as bottled water, dairy- and plant-based drinks, teas, fruit juices, and carbonated beverages—are consumed extensively across all age groups, including vulnerable populations. Consequently, expanding PFAS monitoring to encompass USC compounds in beverages is critical for accurate exposure evaluation and strengthened food safety management. Although comprehensive PFAS analysis incorporating USC compounds have been reported for bottled water and ready-to-drink (RTD) milk products [19,21], PFAS surveillance remains limited for other beverages such as teas and fruit juices. These products are manufactured using ingredients and water sources that may already contain trace PFAS, with further potential contamination through contact with processing equipment, filtration systems, storage tanks, and packaging materials. Currently, no international standards or harmonized maximum residue levels (MRLs) exist for PFAS in beverages. Toxicological concern surrounding PFAS is primarily driven by their persistence, bioaccumulation potential, and associations with adverse health outcomes including developmental effects, immune suppression, dyslipidemia, thyroid disruption, and liver toxicity for several well-studied compounds such as PFOA, PFOS, PFHxS, and PFNA [4]. Consequently, regulatory agencies have established health-based guidance values for selected PFAS. The European Food Safety Authority (EFSA) established a tolerable weekly intake (TWI) of 4.4 ng/kg body weight for the sum of PFOA, PFOS, PFHxS, and PFNA and has called for more comprehensive occurrence data in food products including drinking water, milk, fish, and fruit juice [22]. In the United States, the Food and Drug Administration (FDA) has conducted PFAS surveillance as part of its Total Diet Study, detecting select PFAS in seafood, meat products, and beverages, although no enforceable limits have yet been established for PFAS in food [23]. Meanwhile, the U.S. Environmental Protection Agency (EPA) has finalized maximum contaminant levels (MCLs) for six PFAS in drinking water—including PFOA and PFOS—with health-based limits set at 4 ng/L [24], highlighting the need for equally sensitive detection in food matrices such as beverages. Although toxicological datasets remain more limited for ultrashort-chain PFAS, their extreme persistence, high mobility, and increasing environmental occurrence have raised concern regarding continuous low-level human exposure and the need for expanded occurrence monitoring. In this study, a panel of representative RTD tea and juice products was selected to develop and verify a comprehensive LC-MS/MS method capable of simultaneously analyzing USC and long-chain PFAS. The beverage panel included unsweetened green tea, sweetened black tea, apple juice, a blended berry juice, and orange juice. These selections captured a diverse range of acidity, sugar levels, pigment content, and natural organic matter that could affect PFAS recovery and matrix interference.

Chromatographic analysis of USC PFAS is inherently challenging due to their high polarity, minimal retention on traditional reversed-phase LC columns, and susceptibility to loss during multi-step sample preparation. Conventional LC-MS/MS methods using C18 columns are generally optimized for PFAS with carbon chain lengths of C4 and longer and therefore providing insufficient retention for USC compounds such as TFA. Anion-exchange columns have been explored as an alternative for USC PFAS, although they often suffer from long retention times and broad peak shapes that reduce sensitivity and throughput [7]. More recently, a polar-embedded alkyl phase column has demonstrated optimal performance for balanced retention of USC and long-chain PFAS, enabling their simultaneous determination in water and other matrices [19,21,25]. By adapting this polar-embedded chromatographic approach, the present study aimed to establish a streamlined and universal workflow for comprehensive PFAS determination in a variety of RTD teas and fruit juices. The method targeted 43 analytes encompassing C2–C14 perfluoroalkyl carboxylic acids, C1–C13 perfluoroalkyl sulfonic acids, fluorotelomer carboxylic acids and sulfonic acids, perfluorooctane sulfonamides and sulfonamidoacetic acids, as well as per- and polyfluoroether carboxylic acids and sulfonic acids. The objectives were to: (1) develop a simplified sample preparation procedure that minimizes contamination while enabling accurate quantification; (2) optimize chromatographic conditions to achieve efficient analysis of USC through long-chain PFAS within a short run time; and (3) apply the finalized workflow to assess PFAS contamination across a broader range of commercial beverages. By providing a reliable and robust analytical tool for both ultrashort-chain and long-chain PFAS, this study addresses a critical gap in beverage safety assessment and supports future public health and regulatory efforts for PFAS monitoring in beverage matrices.

## 2. Materials and Methods

### 2.1. Chemicals and Materials

LC/MS-grade acetonitrile used for preparing the organic mobile phase was obtained from Fisher Scientific (Waltham, MA, USA). A second acetonitrile formulation intended for PFAS analysis was purchased from Sigma-Aldrich (St. Louis, MO, USA) and used for preparing standard and sample solutions. Methanol (HPLC grade) was also sourced from Sigma-Aldrich for evaluating sample preparation conditions. Ammonium formate and formic acid (reagent grade) were likewise purchased from Sigma-Aldrich. A PFAS calibration mixture containing 28 analytes (PFAS 28) was supplied by Restek Corporation (Bellefonte, PA, USA). Additional reference materials—including an isotopically labeled mixture (MPFAC-24ES), two multicomponent standards (PFAC-MXG and PFAC-MXI), and individual PFAS standards such as trifluoroacetic acid, perfluoropropanoic acid, sodium trifluoromethanesulfate, sodium perfluoroethanesulfonate, sodium perfluoropropane-sulfonate, sodium perfluoroundecanesulfonate, sodium perfluorododecanesulfonate, sodium perfluorotridecanesulfonate, 3-perfluoropropyl propanoic acid, 3-perfluoropentyl propanoic acid, and 3-perfluoroheptyl propanoic acid—were obtained from Wellington Laboratories (Guelph, ON, Canada). Two isotope solutions, ^13^C_2_-trifluoroacetic acid and ^13^C_3_-perfluoropropanoic acid, were acquired from Cambridge Isotope Laboratories (Andover, MA, USA). The ultrapure deionized water was collected from the Restek Corporation facility for the preparation of standard solutions and aqueous mobile phase. RTD beverage samples were purchased from local grocery stores. For sample preparation, 15 mL polypropylene centrifuge tubes were supplied by VWR International (Radnor, PA, USA). Polypropylene vials (700 µL) and polyethylene caps used for standard and sample preparation were sourced from Waters Corp. (Milford, MA, USA). Ultra IBD Inert column (100 × 2.1 mm, 3 µm) and Ultra IBD column (150 × 2.1 mm, 3 µm) were obtained from Restek Corporation (Bellefonte, PA, USA).

### 2.2. Instrumentation and Chromatographic Method

LC-MS/MS analysis was performed on a Waters Acquity I-Class UPLC system coupled to a Xevo TQ-S triple-quadrupole mass spectrometer. All analytes were optimized under negative electrospray ionization, where precursor ions and their corresponding fragment ions were evaluated to determine the most sensitive transitions. The highest-intensity product ion (quantifier) was used as the primary transition for quantitation, while a secondary transition (qualifier) served as confirmation when available. For several ultrashort- and short-chain compounds such as TFA, PFPrA, PFBA, and PFPeA, only a single product ion was observed, and therefore no qualifier transition could be assigned. HFPO-DA was monitored using the in-source fragmented precursor ion [M-COOH]^−^, which produced stronger signal response under the analytical conditions.

Chromatographic separation of 43 target analytes and 13 quantitative internal standards was achieved using a Restek Ultra IBD Inert column (100 × 2.1 mm, 3.0 µm). To effectively remove background PFAS contamination, an Ultra IBD column (150 × 2.1 mm, 3.0 µm) was installed as a delay column. Mobile Phase A consisted of ultrapure water containing 2 mM ammonium formate and 0.1% formic acid. Mobile Phase B was composed of acetonitrile:water (95:5, *v*/*v*) with the same buffer additives. The gradient program initiated at 50% B, increased linearly to 100% B over 0–7 min, held at 100% B until 10 min, and then returned to 50% B for re-equilibration through 12 min. The column was maintained at 40 °C, with a flow rate of 0.4 mL/min, and 20 µL of sample was injected for each run.

Instrument settings for the mass spectrometer included a capillary voltage of 0.5 kV, desolvation temperature of 550 °C, desolvation gas flow of 800 L/h, cone gas flow of 150 L/h, nebulizer pressure of 7.0 bar, and collision gas flow of 0.20 mL/min. Retention times and individual MRM transitions for all analytes are summarized in Table 1.

### 2.3. Standard and Sample Preparation

A combined standard stock (50 ng/mL) containing all 43 target PFAS was prepared in a 1:1 (*v*/*v*) mixture of acetonitrile and water. Four standards—PFHxS, PFOS, NMeFOSAA, and NEtFOSAA—were supplied as technical-grade materials containing both linear and branched isomers; therefore, reported concentrations reflect the sum of all isomeric forms.

Calibration standards (500 µL) were prepared in polypropylene autosampler vials using a 1:1 mixture of ultrapure water and acetonitrile, covering a concentration range of 1 to 2000 ng/L. Thirteen isotopically labeled PFAS were implemented as quantitative internal standards (QIS; listed in Table 1). Each calibration solution was fortified with 2.5 µL of the QIS working solution (10 ng/mL for each labeled analyte).

For beverage testing, 0.5 mL of sample was aliquoted into 15 mL polypropylene centrifuge tubes, spiked with 5.0 µL of QIS working solution, and mixed with 0.5 mL of acetonitrile by vortexing for 30 s. The tubes were centrifuged at 4000 rpm, and the resulting supernatant was transferred to polypropylene vials for LC-MS/MS analysis.

### 2.4. System Background Control and Blank Monitoring

Because background PFAS contamination can arise from solvents, mobile phases, instrument tubing and seals, and laboratory consumables, multiple control measures were implemented throughout the study. A delay column was installed upstream of the injector to retain instrument-derived PFAS contaminants prior to analytical separation. Previous studies [21] demonstrated that use of a column packed with the same stationary phase as the analytical column (Ultra IBD) effectively separated background contamination peaks, particularly those of long-chain PFAS compounds, from the target analytes. Procedural blanks consisting of ultrapure water processed identically to samples were included with each analytical batch. Instrument blanks were also injected periodically throughout sequences to assess carryover and baseline stability. Background peaks detected in blanks were required to remain below the reporting threshold or below 30% of the analyte response at the lowest calibration level.

## 3. Results

### 3.1. LC-MS/MS Method Development

**Figure 1:**
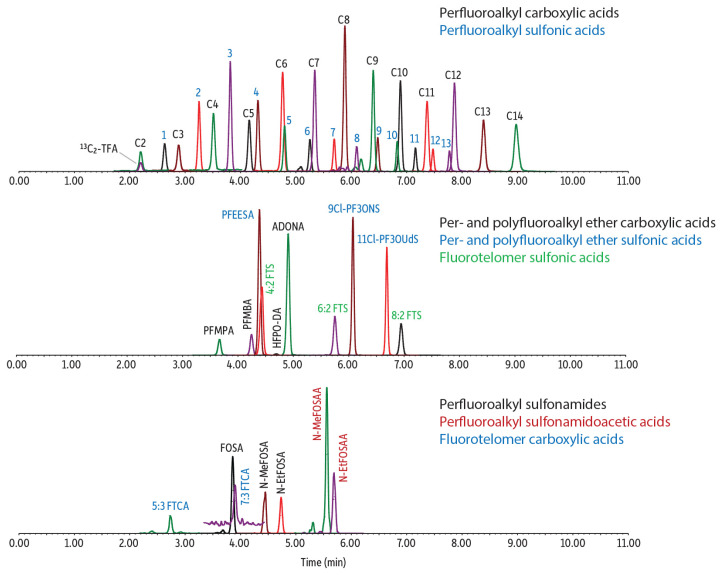
Chromatogram for the analysis of a 500 ng/L fortified blended berry sample on the Ultra IBD Inert column.

Analytical workflows of incorporating USC compounds into comprehensive PFAS analysis have previously been established for diverse water and milk matrices using the polar-embedded alkyl phase Ultra IBD column [19,21]. Earlier work also demonstrated that the inert-coated version of this column provided enhanced detection sensitivity for many PFAS species [19]. Building on these findings, the present study applied the same chromatographic strategy to PFAS determination in beverages, with a focus on evaluating its suitability across the diverse compositions and matrix characteristics of RTD beverages. After examining chromatographic behavior across the different beverage matrices, the LC conditions were refined from the earlier method to improve retention and peak shape for early-eluting compounds. Introducing a consistent and optimized concentration of additives to both the aqueous and organic mobile phases enhanced overall separation performance and also promoted faster elution of long-chain PFAS, thereby shortening the total run time. Figure 1 presents the chromatogram of a fortified blended berry juice analyzed under the finalized LC conditions.

### 3.2. Sample Preparation Method Development

Given the liquid nature of beverage samples, the dilute-and-shoot approach previously applied to water analysis [19] was regarded as suitable for this study. As noted earlier, a simplified sample preparation workflow is essential for minimizing contamination. Background TFA contamination, in particular, poses a significant challenge, as it may be present in solvents, reagents, and common laboratory materials. To mitigate this, those pre-cleaned pipette tips, HPLC vials, and centrifuge tubes—previously identified as low-contamination materials—were used throughout the sample preparation process.

In previous studies, methanol was employed as the dilution solvent for water samples [19], whereas acetonitrile was found to be more effective for milk sample preparation [21]. Accordingly, both solvents were evaluated for beverage sample dilution to assess their impact on chromatographic performance. For both blank and fortified samples, long-chain PFAS exhibited stronger and more consistent detection signals when samples were diluted 2-fold with acetonitrile compared to methanol. Based on these results, acetonitrile was selected as the diluent for beverage sample preparation.

A variety of tea and fruit juice beverages were tested to evaluate the performance of this straightforward dilute-and-shoot procedure. After dilution, centrifugation was required to remove suspended particles or pulp and to obtain a clean supernatant for LC-MS/MS analysis. Injection volumes up to 50 µL were examined to assess their impact on chromatographic performance. Some beverages, particularly apple and orange juices, showed reduced retention and broader peak shapes for early-eluting analytes at higher injection volumes. Ultimately, a 20 µL injection volume was identified as optimal, providing improved detection sensitivity while maintaining stable chromatographic performance across all beverages. Additionally, all tested orange juice samples exhibited stronger matrix effects, leading to poor peak shapes for early-eluting compounds. To address this, orange juice samples required an initial 2-fold dilution with water prior to the acetonitrile dilute-and-shoot step.

### 3.3. Assessment of Matrix Effects

For matrix effect evaluation, five representative RTD beverages—unsweetened green tea, sweetened black tea, apple juice, a red berry juice blend, and orange juice—were selected to cover a broad variability of acidity, sugar content, pigment levels, and natural organic matter that may influence MS ionization behavior. The dilute-and-shoot procedure was used to prepare blank sample solutions for each beverage, which were then fortified at 100 ng/L for LC-MS/MS analysis. Analyte peak areas were compared with those of a 100 ng/L standard solution to assess matrix effects. Given the high incurred TFA levels observed in all beverage samples, ^13^C_2_-TFA was employed as a surrogate to assess the matrix effect for TFA. This isotope was confirmed to produce the MS response that closely mirrored the native TFA. As summarized in Supplementary Document Table S1, ultrashort-chain and short-chain perfluoroalkyl carboxylic acids exhibited pronounced signal suppression across all beverage types. Similar suppression trends were observed for fluorotelomer carboxylic acids, perfluorooctane sulfonamides, and sulfonamidoacetic acids. In contrast, perfluoroalkyl sulfonic acids were generally less affected by matrix-induced signal suppression. These results demonstrate that beverage matrices impose substantial and variable matrix effects on PFAS detection, emphasizing the need to include isotopically labeled PFAS as quantitative internal standards (QIS) to adequately correct for these interferences and ensure accurate quantification.

### 3.4. Evaluation of Method Accuracy and Precision

To evaluate method accuracy and precision, five representative beverage samples were fortified with native analytes at 2, 4, 10, 20, 100, and 500 ng/L. Isotopically labeled ^13^C_2_-TFA was included as a surrogate for determining TFA recovery, and a mixture of 19 mass-labeled PFAS was added to each fortified sample as QIS. Three analytical batches were conducted on separate days, with three replicates per fortified concentration in each batch, resulting in a total of nine replicates for each fortification level. Various incurred PFAS were present in all beverages, and their background concentrations were subtracted from the measured values in fortified samples to calculate recoveries.

In developing a universal quantification method for diverse beverage matrices, significant effort was dedicated to identifying suitable pairings of isotopically labeled QIS with native PFAS to achieve consistent and acceptable recoveries. Given the wide variability in matrix effects across the five representative beverages, establishing a single QIS-native compound pairing for all samples posed a considerable challenge. Through extensive testing, 13 out of 19 available isotopes were ultimately selected as QIS, ensuring that the same QIS-native analyte pairings could be applied consistently across all beverage types (see Table 1). This approach enabled reliable correction for matrix effects and accurate quantification of PFAS in a broad range of beverages.

Using quadratic regression with 1/x weighting, all analytes demonstrated strong linearity, with r^2^ values greater than 0.995 and deviations below 30%. Calibration ranges were set as follows: 1–1000 ng/L or 2–1000 ng/L for most analytes, 10–1000 ng/L for HFPO-DA, 10–2000 ng/L for TFA, and 4–1000 ng/L for the remaining compounds (Supplementary Document Table S2). The method’s limit of quantitation (LOQ) was defined as the lowest fortified concentration that could be quantified with acceptable accuracy. The limit of detection (LOD) was estimated from fortified beverage samples based on a peak signal-to-noise ratio of 3 (Supplementary Document Table S2).

The average recovery and relative standard deviation (RSD) values are summarized in Supplementary Document Table S3. Recovery values were in the range of 75.3–114% in green tea, 71.1–118% in black tea, 83.6–117% in apple juice, 87.6–121% in berry juice, and 83.9–122% in orange juice. Satisfactory method precision was demonstrated with %RSD values within 12% for all beverage samples. Overall, depending on the beverage type, multiple analytes could be accurately quantified with an LOQ of 2 ng/L, except for orange juice, which required additional dilution upon sample preparation. Certain compounds—such as TFA (monitored using ^13^C_2_-TFA), HFPO-DA, fluorotelomer carboxylic acids, perfluorooctane sulfonamides, and sulfonamidoacetic acids—exhibited higher LOQs of 10 or 20 ng/L due to relatively lower MS detection sensitivity or stronger matrix interferences, varying by beverage type. Nevertheless, the majority of 43 targeted PFAS were accurately quantifiable at 2 or 4 ng/L, demonstrating that the developed analytical workflow provides a simple, robust, and reliable approach for PFAS determination across diverse beverage matrices.

### 3.5. Screening of PFAS in an Expanded Selection of Commercial Beverages

The verified analytical workflow was subsequently applied to profile PFAS in a total of 23 commercial beverages purchased from local grocery stores. In addition to unsweetened and sweetened green and black teas, several fruit-flavored black teas were included in the screening. For fruit juices, the evaluation was expanded to incorporate a broader variety, including grape, cranberry, pineapple, and multiple mixed-fruit juices. All samples were analyzed in duplicate. Because full accuracy and precision assessments were not performed for these additional beverage types, the reported concentrations should be regarded as estimates. Nevertheless, based on the accuracy results obtained from the five representative beverages, the quantification performance for these additional samples is expected to be reasonably reliable.

The results revealed that ultrashort-chain and short-chain PFAS constituted the primary contributors to PFAS contamination across the beverage samples (Supplementary Document Table S4). As expected, all beverages contained TFA at substantially higher concentrations than other PFAS. For several samples, TFA levels exceeded the upper limit of the calibration range, requiring additional dilution to obtain accurate quantification. The highly acidic TFMS was also commonly presented in beverages, with concentrations ranging from below 2 ng/L to more than 200 ng/L. Other ultrashort-chain PFAS were also identified, with PFPrA detected in green tea and several fruit juice samples, whereas PFPrS was consistently observed across all tea beverages. Among short-chain compounds, PFBA, PFBS, and PFPeS were detected in several tea and fruit juice samples, with PFPeS occurring widely in both green and black teas. Long-chain PFAS were rarely observed, with PFOA and PFOS detected in only a few samples. Interestingly, PFTrDA appeared to be more prevalent across the tea beverages. A polyfluoroether sulfonic acid compound, PFEESA, was the sole alternative PFAS detected in a few beverages. Overall, ultrashort-chain compounds were identified as the predominant PFAS contaminants in both tea and fruit juice beverages.

## 4. Discussion

### 4.1. Optimization of Analytical Workflows

The success of the inert-coated mixed-mode alkyl phase column in this study aligns with previous findings in water and milk matrices [19,21], confirming its versatility for complex food-grade liquids. The decision to use a 20 µL injection volume was a critical compromise; while higher volumes theoretically increase sensitivity, the observed peak broadening in apple and orange juices suggests that matrix constituents (sugars and organic acids) interfere with the chromatographic performance of early-eluting polar analytes. The requirement for an additional 2-fold aqueous dilution for orange juice further underscores the unique challenge of high-citrate or high-pulp matrices in “dilute-and-shoot” workflows.

Regarding sample preparation, the superiority of acetonitrile over methanol for beverage dilution mirrors results previously seen in milk [21]. This suggests that acetonitrile may more effectively precipitate or solubilize specific matrix components that otherwise interfere with the detection of long-chain PFAS.

### 4.2. Interpretation of Matrix Interference and Quantification

The pronounced signal suppression observed for ultrashort-chain and short-chain perfluoroalkyl carboxylic acids across all five representative beverages highlights the chemical complexity of RTD teas and juices. The variability in MS ionization behavior—likely driven by differing levels of pigments and natural organic matter—justifies our rigorous selection of specific QIS and native analyte pairings. Using ^13^C_2_-TFA as a surrogate for native TFA proved essential, as high background levels of TFA in commercial samples would otherwise make recovery assessment impossible. From a practical implementation perspective, beverage matrices with elevated sugar, organic acid, pulp, or pigment content should be screened during method transfer using post-spike recovery checks. For challenging matrices such as orange juice, additional pre-dilution with water followed by acetonitrile precipitation proved effective in reducing matrix suppression and improving chromatographic peak shape. Therefore, a tiered preparation strategy is recommended: (i) direct dilute-and-shoot for clear beverages, (ii) water pre-dilution for highly acidic or viscous juices, and (iii) isotope dilution quantification for all matrices.

### 4.3. Implications of PFAS Occurrence in Beverages

The predominance of USC compounds (TFA, TFMS, PFPrA, PFPrS) over long-chain species (PFOA, PFOS) in our commercial screening reflects a broader shift in the environmental PFAS profile. Beyond their overall predominance, several recurring occurrence patterns were observed across the beverage categories examined. First, ultrashort-chain PFAS consistently represented the major contributors to total PFAS burdens in both tea and juice beverages, whereas legacy long-chain PFAS were detected only infrequently. Second, TFA was universally present and commonly occurred at concentrations substantially higher than those of other PFAS. Third, tea beverages showed more frequent detection of PFPrS, PFPeS, and PFTrDA than fruit juices, suggesting possible differences associated with source water, raw materials such as tea leaves, or beverage processing and packaging materials. These recurring profiles indicate that contamination patterns in beverages may be shifting toward highly mobile short-chain and ultrashort-chain PFAS rather than the historically emphasized long-chain compounds. The high levels of TFA and the frequent detection of PFPrA and PFPrS suggest that these beverages may be an overlooked contributor to total dietary PFAS intake. While long-chain compounds are often the focus of regulatory concern, our results indicate that a “comprehensive” assessment of human exposure via the food chain is incomplete without the inclusion of these highly mobile, ultrashort-chain species.

Although the present study was not designed as a dietary exposure assessment, the occurrence data generated here indicate that ready-to-drink beverages may contribute to cumulative PFAS intake, particularly for highly prevalent ultrashort-chain compounds such as TFA and TFMS. Quantitative estimation of population exposure will require broader occurrence surveys combined with beverage consumption statistics across age groups and body weights. In addition, health-based guidance values remain unavailable for many ultrashort-chain PFAS, limiting formal risk characterization at present. Nevertheless, the analytical workflow established here provides a practical tool for generating the occurrence datasets needed for future exposure and risk assessments.

### 4.4. Comparison with Existing Methods

Compared with many existing PFAS methods for beverages and foods, the present workflow offers three distinct advantages. First, conventional methods often emphasize C4 and longer PFAS and do not adequately retain C1–C3 analytes such as TFA and TFMS. Second, many published methods rely on SPE or multi-step cleanup procedures that increase labor and contamination risk. Third, run times frequently exceed 15–20 min. In contrast, the present method enables simultaneous determination of C1–C14 PFAS using a simple dilute-and-shoot workflow with a 12 min chromatographic cycle and low-ng/L quantification limits for most analytes. These characteristics make the method suitable for higher-throughput surveillance applications.

## 5. Conclusions

This study established a simple, reliable, and broadly applicable analytical workflow for the comprehensive determination of ultrashort-chain to long-chain PFAS in diverse ready-to-drink tea and fruit juice beverages. Application of the verified workflow to an expanded set of commercial beverages revealed that ultrashort-chain PFAS, particularly TFA and TFMS, were the predominant contaminants, with additional contributions from PFPrA, PFPrS, and certain short-chain PFAS. Long-chain PFAS were only sporadically detected across the beverages analyzed. Overall, this study demonstrates the feasibility and practicality of incorporating ultrashort-chain PFAS into routine monitoring of beverages. The workflow provides a robust foundation for future surveillance of PFAS contamination in commercial drink products and supports ongoing efforts to better characterize PFAS exposure through food and beverages.

## Figures and Tables

**Table 1:** MS ion transitions, parameters, and chromatographic retention time of analytes.

Compounds	Retention	Precursor Ion	Product Ions *^a^*	Cone (V)	Collision (V)	Quantification
	Time (min)					Internal Standard
***Target Analytes***						
**Perfluoroalkyl carboxylic acids**						
Trifluoroacetic acid (TFA)	2.20	113.03 [M-H]^−^	69.01	10	10	^13^C_3_-PFPrA
Perfluoropropanoic acid (PFPrA)	2.88	162.97 [M-H]^−^	119.02	10	8	^13^C_3_-PFPrA
Perfluorobutanoic acid (PFBA)	3.51	213.03 [M-H]^−^	168.98	14	8	^13^C_4_-PFBA
Perfluoropentanoic acid (PFPeA)	4.16	262.97 [M-H]^−^	218.97	2	6	^13^C_5_-PFPeA
Perfluorohexanoic acid (PFHxA)	4.76	313.10 [M-H]^−^	268.97/118.99	2	8/20	^13^C_5_-PFHxA
Perfluoroheptanoic acid (PFHpA)	5.34	363.16 [M-H]^−^	319.09/169.06	8	10/18	^13^C_4_-PFHpA
Perfluorooctanoic acid (PFOA)	5.88	413.10 [M-H]^−^	368.96/168.90	2	10/16	^13^C_8_-PFOA
Perfluorononanoic acid (PFNA)	6.40	463.10 [M-H]^−^	419.01/219.02	4	10/16	^13^C_9_-PFNA
Perfluorodecanoic acid (PFDA)	6.89	513.17 [M-H]^−^	469.16/219.06	4	12/16	^13^C_6_-PFDA
Perfluoroundecanoic acid (PFUnA)	7.37	563.23 [M-H]^−^	519.24/269.07	6	12/18	^13^C_2_-PFTeDA
Perfluorododecanoic acid (PFDoA)	7.86	613.23 [M-H]^−^	569.19/169.06	8	12/26	^13^C_2_-PFTeDA
Perfluorotridecanoic acid (PFTrDA)	8.38	663.23 [M-H]^−^	619.21/169.06	8	14/28	^13^C_2_-PFTeDA
Perfluorotetradecanoic acid (PFTeDA)	8.98	712.67 [M-H]^−^	668.69/168.94	10	12/26	^13^C_2_-PFTeDA
**Perfluoroalkyl sulfonic acids**						
Trifluoromethanesulfonic acid (TFMS)	2.63	148.97 [M-H]^−^	79.93/98.92	62	13/18	^13^C_3_-PFBS
Perfluoroethanesulfonic acid (PFEtS)	3.25	198.90 [M-H]^−^	79.92/98.91	38	22/22	^13^C_3_-PFHxS
Perfluoropropanesulfonic acid (PFPrS)	3.81	248.97 [M-H]^−^	79.92/98.91	2	24/24	^13^C_5_-PFPeA
Perfluorobutanesulfonic acid (PFBS)	4.32	298.97 [M-H]^−^	79.97/98.89	2	26/26	^13^C_3_-PFBS
Perfluoropentanesulfonic acid (PFPeS)	4.79	349.10 [M-H]^−^	79.98/98.98	6	32/30	^13^C_3_-PFHxS
Perfluorohexanesulfonic acid (PFHxS)	5.25	398.90 [M-H]^−^	79.97/98.89	56	32/34	^13^C_3_-PFHxS
Perfluoroheptanesulfonic acid (PFHpS)	5.69	449.17 [M-H]^−^	79.98/98.97	4	42/38	^13^C_3_-PFHxS
Perfluorooctanesulfonic acid (PFOS)	6.11	499.03 [M-H]^−^	79.92/98.90	8	40/40	^13^C_8_-PFOS
Perfluorononanesulfonic acid (PFNS)	6.48	549.10 [M-H]^−^	79.92/98.83	12	42/40	^13^C_8_-PFOS
Perfluorodecanesulfonic acid (PFDS)	6.83	599.17 [M-H]^−^	79.98/98.83	8	44/46	^13^C_8_-PFOS
Perfluoroundecanesulfonic acid (PFUdS)	7.16	648.73 [M-H]^−^	79.94/98.94	38	50/44	^13^C_8_-PFOS
Perfluorododecanesulfonic acid (PFDoS)	7.47	698.77 [M-H]^−^	79.95/98.94	10	60/44	^13^C_8_-PFOS
Perfluorotridecanesulfonic acid (PFTrDS)	7.77	748.73 [M-H]^−^	79.94/98.94	8	76/52	^13^C_8_-PFOS
**Fluorotelomer sulfonic acids**						
1H,1H,2H,2H-Perfluorohexane sulfonic acid (4:2 FTS)	4.44	327.10 [M-H]^−^	307.08/80.83	50	18/24	^13^C_3_-PFHxS
1H,1H,2H,2H-Perfluorooctane sulfonic acid (6:2 FTS)	5.75	427.17 [M-H]^−^	407.18/80.71	2	22/32	^13^C_8_-PFOA
1H,1H,2H,2H-Perfluorodecane sulfonic acid (8:2 FTS)	6.94	527.17 [M-H]^−^	507.16/80.83	66	26/32	^13^C_3_-PFHxS
**Fluorotelomer carboxylic acids**						
3-perfluoropentyl propanoic acid (5:3 FTCA)	2.73	340.93 [M-H]^−^	216.96/236.93	2	24/14	^13^C_3_-PFPrA
3-perfluoroheptyl propanoic acid (7:3 FTCA)	3.92	440.90 [M-H]^−^	336.88/316.91	20	12/22	^13^C_3_-PFBA
**Perfluoroalkyl sulfonamides**						
Perfluorooctanesulfonamide (FOSA)	3.87	498.17 [M-H]^−^	77.97/477.76	8	28/26	^13^C_8_-FOSA
N-methyl perfluorooctanesulfonamide (NMeFOSA)	4.45	511.77 [M-H]^−^	168.95/218.91	2	26/24	^13^C_5_-PFPeA
N-ethyl perfluorooctanesulfonamide (NEtFOSA)	4.73	525.83 [M-H]^−^	168.96/218.92	10	26/24	^13^C_5_-PFHxA
**Perfluoroalkyl sulfonamidoacetic acids**						
N-methyl perfluorooctanesulfonamidoacetic acid (NMeFOSAA)	5.55	570.20 [M-H]^−^	419.17/483.16	46	20/14	^13^C_3_-PFPrA
N-ethyl perfluorooctanesulfonamidoacetic acid (NEtFOSAA)	5.68	584.20 [M-H]^−^	419.18/483.11	6	20/16	^13^C_3_-PFPrA
**Per- and Polyfluoroether carboxylic acids**						
Perfluoro-3-methoxypropanoic acid (PFMPA)	3.68	228.93 [M-H]^−^	84.97/198.94	10	10/14	^13^C_4_-PFBA
Perfluoro-4-methoxybutanoic acid (PFMBA)	4.24	278.87 [M-H]^−^	84.96/234.93	8	10/6	^13^C_5_-PFHxA
Hexafluoropropylene oxide dimer acid (HFPO-DA)	4.68	285.03 [M-COOH]^−^	169.02/185.02	2	6/16	^13^C_3_-PFHxS
4,8-Dioxa-3H-perfluorononanoic acid (ADONA)	4.90	376.90 [M-H]^−^	250.93/84.97	22	12/26	^13^C_4_-PFHpA
**Per- and Polyfluoroether sulfonic acids**						
Perfluoro(2-ethoxyethane)sulfonic acid (PFEESA)	4.09	314.83 [M-H]^−^	134.94/83.01	4	22/16	^13^C_3_-PFHxS
9-Chlorohexadecafluoro-3-oxanonane-1-sulfonic acid (9Cl-PF3ONS)	6.08	530.78 [M-H]^−^	350.85/82.96	12	26/24	^13^C_2_-PFTeDA
11-Chloroeicosafluoro-3-oxaundecane-1-sulfonic acid (11Cl-PF3OUdS)	6.69	630.78 [M-H]^−^	450.80/82.95	8	26/32	^13^C_2_-PFTeDA
***Surrogate***						
^13^C_2_-TFA	2.20	114.90 [M-H]^−^	69.95	14	8	^13^C_3_-PFPrA
***Quantification Internal Standards***						
^13^C_3_-PFPrA	2.88	165.97 [M-H]^−^	120.96	10	11	
^13^C_4_-PFBA	3.51	217.03 [M-H]^−^	171.98	2	8	
^13^C_5_-PFPeA	4.16	267.97 [M-H]^−^	222.99	2	6	
^13^C_5_-PFHxA	4.76	318.03 [M-H]^−^	272.93	2	7	
^13^C_4_-PFHpA	5.34	366.90 [M-H]^−^	321.93	2	10	
^13^C_8_-PFOA	5.88	420.97 [M-H]^−^	375.94	2	10	
^13^C_9_PFNA	6.40	471.97 [M-H]^−^	426.87	4	12	
^13^C_6_-PFDA	6.89	518.90 [M-H]^−^	473.87	4	13	
^13^C_2_-PFTeDA	8.98	714.78 [M-H]^−^	669.80	8	14	
^13^C_3_-PFBS	4.32	301.97 [M-H]^−^	79.97	2	28	
^13^C_3_-PFHxS	5.25	401.90 [M-H]^−^	79.97	2	36	
^13^C_8_-PFOS	6.11	506.84 [M-H]^−^	79.97	4	42	
^13^C_8_-FOSA	3.87	505.91 [M-H]^−^	77.95	4	32	

*^a^* Quantifier Ion/Qualifier Ion.

## Data Availability

The original contributions presented in this study are included in the article/Supplementary Material. Further inquiries can be directed to the corresponding author.

## Supplementary Materials

The following supporting information can be downloaded at: https://www.mdpi.com/article/10.3390/toxics14050422/s1, Table S1: Assessment of matrix effects; Table S2: Linearity and limit of detection; Table S3: Accuracy and precision for the analysis of fortified beverage samples; Table S4: Measurement of PFAS in commercial beverage samples.
