# Supplementary material for: Comprehensive Per- and Polyfluorinated Substances Profiling in Beverages: Simultaneous Quantification of Ultrashort-Chain to Long-Chain Compounds in Ready-to-Drink Teas and Fruit Juices
Source: Toxics. 2026 May 12;14(5):422. doi: 10.3390/toxics14050422 (PMC13211061; doi:10.3390/toxics14050422)
Supplement: Supplementary file 1 [file toxics-14-00422-s001.zip › toxics-4286854-supplementary.pdf]

Supplementary Materials

Title

Comprehensive per- and polyfluorinated substances profiling in beverages: simultaneous quantification of ultrashort-chain to long-chain compounds in ready-to-drink teas and fruit juices

Authors

Shun-Hsin Liang (shun-hsin.liang@restek.com)

Justin A. Steimling (justin.steimling@restek.com)

Affiliation (all authors)

Restek Corporation, 110 Benner Circle, Bellefonte, PA 16823, USA

Corresponding Author

Shun-Hsin Liang, Ph.D.

Email: shun-hsin.liang@restek.com

Telephone: 1-814-470-1022

Fax: 1-814-353-9067

Address: 110 Benner Circle, Bellefonte, PA 16823, USA

Table S1. Assessment of matrix effects

Analytes	Peak Area Ratio, % (Fortified Sample)/(Standard Solution)				
	Green Tea	Black Tea	Apple Juice	Berry Juice	Orange Juice
$^{13}C_2$-TFA	33.6	30.0	26.7	31.2	28.3
PFPrA	45.7	35.8	30.1	36.5	33.5
PFBA	48.1	37.9	33.7	41.8	39.7
PFPeA	54.1	43.4	43.8	52.2	45.8
PFHxA	68.2	54.8	60.5	62.9	62.5
PFHpA	54.9	39.9	47.0	52.3	47.9
PFOA	76.5	60.6	69.9	72.8	57.9
PFNA	83.9	72.7	79.5	84.8	72.9
PFDA	77.2	61.1	66.8	74.0	69.3
PFUnA	90.9	81.5	86.3	91.8	88.7
PFDoA	86.5	84.1	90.5	89.0	96.0
PFTrDA	85.2	79.0	82.6	85.2	86.2
PFTeDA	86.6	81.5	87.8	89.1	96.3
TFMS	78.5	74.7	71.7	78.1	73.8
PFEtS	94.9	97.6	90.8	93.7	92.2
PFPrS	89.9	90.9	88.5	91.3	87.1
PFBS	69.0	60.7	65.0	71.6	66.7
PFPeS	86.0	86.7	81.9	84.4	82.7
PFHxS	74.3	69.0	83.2	88.5	74.5
PFHpS	85.8	77.9	82.2	85.9	85.4
PFOS	98.0	95.7	99.8	101	100
PFNS	98.0	94.1	105	103	98.0
PFDS	89.4	77.4	89.7	86.0	90.8
PFUdS	92.0	87.4	94.9	93.9	97.1
PFDoS	88.9	86.6	93.7	93.5	95.8
PFTrDS	89.0	87.3	94.6	91.5	90.3
4:2 FTS	80.5	51.2	76.6	70.4	60.7
6:2 FTS	72.0	47.7	66.0	68.8	39.0
8:2 FTS	84.7	69.3	86.3	79.2	86.7
5:3 FTCA	33.0	34.9	27.3	41.0	28.7
7:3 FTCA	38.5	24.3	31.5	42.6	40.2
FOSA	39.8	24.9	33.1	40.5	38.7
NMeFOSA	48.9	36.5	41.8	53.4	52.6
NEtFOSA	54.0	44.3	53.5	61.8	67.7
NMeFOSAA	38.3	25.8	31.5	49.6	22.6
NEtFOSAA	43.6	32.9	37.5	46.4	23.8
PFMPA	43.3	29.8	34.6	40.4	35.4
PFMBA	68.0	54.1	57.9	61.8	57.2
HFPO-DA	76.2	51.9	83.2	87.3	79.3
ADONA	61.5	48.3	55.8	61.9	57.1
PFEESA	77.3	71.8	77.8	79.8	77.2
9Cl-PF3ONS	87.0	78.2	87.4	90.7	89.8
11Cl-PF3OUdS	90.6	81.2	92.2	92.6	88.7

Table S2. Linearity and limit of detection

Analytes	Linearity Range[1] (ng/L)	LOD (ng/L)[2]				
		Green Tea	Black Tea	Apple Juice	Berry Juice	Orange Juice
TFA[3]	10 - 2000	1.11	1.85	1.76	1.71	1.76
PFPrA	1 - 1000	1.20	2.40	1.00	0.86	2.20
PFBA	1 - 1000	1.81	1.90	0.92	1.00	1.09
PFPeA	1 - 1000	1.36	1.50	1.35	0.83	1.44
PFHxA	1 - 1000	1.09	1.42	0.83	0.86	1.30
PFHpA	1 - 1000	0.52	0.81	0.43	0.36	0.72
PFOA	1 - 1000	1.03	1.10	0.52	0.55	1.25
PFNA	1 - 1000	1.25	1.33	0.48	0.51	1.35
PFDA	1 - 1000	0.71	1.00	0.44	0.45	0.71
PFUnA	1 - 1000	0.74	1.21	0.41	0.40	1.14
PFDoA	1 - 1000	0.68	0.73	0.26	0.34	0.87
PFTrDA	1 - 1000	0.57	0.82	0.27	0.28	0.71
PFTeDA	1 - 1000	0.41	0.52	0.27	0.26	0.44
TFMS	1 - 1000	0.48	0.67	0.42	0.32	0.60
PFEtS	1 - 1000	0.38	0.50	0.33	0.35	0.53
PFPrS	1 - 1000	0.39	0.73	0.27	0.27	0.79
PFBS	1 - 1000	0.62	0.65	0.39	0.30	0.69
PFPeS	1 - 1000	0.33	0.63	0.31	0.23	0.55
PFHxS	1 - 1000	0.78	0.59	0.90	0.79	0.97
PFHpS	1 - 1000	0.81	0.92	0.58	0.57	0.53
PFOS	1 - 1000	1.12	1.83	1.15	1.28	1.80
PFNS	1 - 1000	0.95	1.12	0.46	0.51	1.23
PFDS	1 - 1000	0.91	1.11	0.47	0.96	1.13
PFUdS	1 - 1000	0.50	0.55	0.71	0.78	0.77
PFDoS	1 - 1000	0.45	0.43	0.54	0.56	0.85
PFTrDS	1 - 1000	0.80	0.71	0.70	0.71	0.80
4:2 FTS	1 - 1000	0.54	0.41	0.38	0.44	0.63
6:2 FTS	1 - 1000	0.75	1.00	1.49	1.57	1.60
8:2 FTS	1 - 1000	0.46	0.63	0.50	0.49	0.85
5:3 FTCA	4 - 1000	6.67	7.50	11.72	7.50	10.00
7:3 FTCA	4 - 1000	15.00	12.00	12.71	15.00	8.33
FOSA	2 - 1000	2.00	2.11	1.58	1.12	2.31
NMeFOSA	2 - 1000	5.00	5.25	3.11	3.13	4.29
NEtFOSAA	2 - 1000	7.10	8.25	2.66	4.50	6.00
NMeFOSAA	4 - 1000	8.21	6.64	5.77	7.50	10.00
NEtFOSAA	4 - 1000	6.98	5.24	3.00	7.73	6.67
PFMPA	1 - 1000	1.09	1.33	1.00	1.36	1.42
PFMBA	1 - 1000	0.78	0.91	0.73	0.65	1.20
HFPO-DA	10 - 1000	4.29	11.20	4.62	4.29	12.5
ADONA	1 - 1000	0.45	0.42	0.48	0.41	0.91
PFEESA	1 - 1000	0.31	0.31	0.18	0.16	0.34
9Cl-PF3ONS	1 - 1000	0.50	0.57	0.29	0.39	0.55
11Cl-PF3OUdS	1 - 1000	0.43	0.25	0.20	0.21	0.30

[1] Calibration range of standard solutions

[2] Concentration in beverage samples

[3] LOD of TFA was determined with mass-labeled $^{13}C_2$-TFA

Table S3. Accuracy and precision for the analysis of fortified beverage samples

Green Tea:

Analytes	Average Recovery (RSD, %), n=9					
	Green Tea					
	Fortified Concentration (ng/L)					
	2	4	10	20	100	500
$^{13}C_2$-TFA	-	-	-	101 (6.01)	97.8 (8.72)	113 (3.76)
PFPrA	-	105 (6.54)	105 (5.99)	108 (3.90)	107 (2.95)	111 (4.51)
PFBA	-	107 (5.66)	98.0 (5.67)	102 (7.19)	107 (4.64)	110 (2.45)
PFPeA	-	98.2 (7.18)	97.6 (8.27)	94.9 (8.82)	104 (7.73)	97.8 (4.89)
PFHxA	-	106 (5.53)	108 (3.28)	105 (7.67)	113 (3.28)	112 (5.54)
PFHpA	95.3 (8.89)	93.4 (8.32)	94.8 (7.10)	98.5 (3.49)	106 (2.38)	90.2 (2.60)
PFOA	-	109 (8.46)	107 (7.81)	109 (7.91)	104 (8.73)	109 (6.47)
PFNA	-	106 (7.63)	110 (5.85)	111 (6.41)	105 (6.69)	109 (3.72)
PFDA	109 (3.55)	94.5 (7.33)	89.5 (7.12)	99.1 (4.35)	100 (4.29)	90.1 (5.56)
PFUnA	110 (6.51)	106 (8.12)	98.8 (7.02)	107 (5.46)	114 (4.04)	109 (5.62)
PFDoA	109 (5.62)	110 (6.66)	103 (6.74)	109 (4.25)	104 (5.74)	101 (8.09)
PFTrDA	99.0 (8.03)	103 (8.56)	107 (8.41)	114 (4.09)	107 (7.30)	100 (8.00)
PFTeDA	113 (7.51)	105 (3.06)	115 (3.26)	106 (7.68)	108 (7.45)	109 (5.41)
TFMS	-	-	105 (4.55)	108 (3.03)	106 (3.74)	111 (3.47)
PFEtS	104 (5.11)	98.2 (8.56)	99.9 (5.89)	97.4 (6.70)	108 (6.50)	107 (6.73)
PFPrS	102 (6.95)	100 (3.67)	107 (5.77)	99.6 (7.01)	104 (6.67)	98.0 (10.1)
PFBS	113 (3.63)	108 (5.07)	110 (5.95)	102 (8.78)	113 (2.56)	104 (4.80)
PFPeS	103 (8.22)	103 (4.62)	102 (2.89)	106 (3.80)	108 (3.27)	112 (8.41)
PFHxS	112 (8.41)	103 (9.71)	98.5 (4.13)	99.5 (7.79)	105 (3.03)	104 (4.54)
PFHpS	111 (5.52)	97.7 (6.31)	99.0 (8.59)	98.3 (9.38)	105 (5.27)	88.7 (6.48)
PFOS	-	100 (8.90)	104 (5.40)	100 (9.13)	111 (4.76)	104 (5.18)
PFNS	-	103 (8.62)	107 (5.07)	113 (5.69)	111 (5.45)	105 (4.31)
PFDS	-	103 (10.2)	100 (9.92)	109 (6.48)	112 (4.47)	100 (6.20)
PFUdS	109 (9.79)	108 (6.41)	103 (7.23)	109 (4.61)	104 (5.29)	100 (2.91)
PFDoS	100 (9.44)	106 (8.64)	107 (7.05)	109 (6.32)	108 (7.18)	107 (3.77)
PFTrDS	-	99.7 (10.0)	98.4 (10.3)	103 (4.11)	110 (7.00)	108 (5.14)
4:2 FTS	102 (10.7)	105 (6.42)	97.0 (8.35)	97.2 (6.37)	110 (5.97)	107 (6.41)
6:2 FTS	113 (3.95)	107 (6.25)	105 (9.47)	100 (9.47)	106 (5.97)	98.5 (7.66)
8:2 FTS	111 (6.32)	105 (10.6)	97.6 (10.7)	101 (8.33)	103 (4.80)	89.4 (9.41)
FOSA	-	-	104 (9.57)	91.8 (10.6)	101 (10.0)	93.4 (7.50)
NMeFOSA	-	-	109 (8.85)	102 (9.02)	96.4 (9.18)	89.0 (6.37)
NEtFOSA	-	-	-	104 (8.60)	107 (6.61)	95.6 (8.79)
5:3 FTCA	-	-	-	104 (8.59)	99.8 (8.23)	86.6 (9.52)
7:3 FTCA	-	-	-	95.1 (4.81)	98.8 (5.82)	86.0 (6.81)
NMeFOSAA	-	-	-	92.9 (11.0)	87.9 (11.5)	75.3 (10.4)
NEtFOSAA	-	-	-	92.2 (10.4)	95.9 (8.36)	83.7 (10.0)
PFMPA		95.7 (5.69)	96.3 (8.57)	94.8 (4.06)	107 (4.69)	104 (5.62)
PFMBA	103 (7.98)	101 (8.80)	106 (6.65)	109 (7.77)	109 (5.97)	113 (3.08)
HFPO-DA	-	-	-	105 (5.97)	100 (9.22)	103 (4.26)
ADONA	109 (7.76)	104 (9.97)	104 (6.68)	102 (9.77)	112 (3.25)	105 (2.95)
PFEESA	105 (6.83)	100 (8.27)	101 (8.66)	101 (8.66)	110 (4.99)	106 (6.19)
9Cl-PF3ONS	110 (5.62)	107 (7.20)	101 (6.31)	112 (5.55)	111 (5.64)	93.0 (2.54)
11Cl-PF3OUdS	112 (6.11)	111 (4.29)	105 (5.28)	108 (6.78)	110 (4.00)	94.6 (1.68)

Black Tea:

Analytes	Average Recovery (RSD, %), n=9					
	Black Tea					
	Fortified Concentration (ng/L)					
	2	4	10	20	100	500
$^{13}C_2$-TFA	-	-	-	99.4 (5.47)	91.8 (3.33)	95.0 (4.11)
PFPrA	-	93.0 (7.52)	103 (9.72)	99.1 (7.80)	115 (5.92)	110 (2.92)
PFBA	-	98.6 (7.36)	100 (9.20)	95.4 (7.10)	110 (6.93)	106 (2.97)
PFPeA	-	106 (9.50)	92.0 (7.30)	95.1 (9.03)	108 (4.32)	90.4 (3.90)
PFHxA	-	107 (8.90)	101 (9.22)	100 (6.33)	112 (3.85)	103 (4.62)
PFHpA	103 (7.55)	99.6 (8.09)	91.5 (9.38)	92.8 (5.09)	99.8 (3.77)	85.7 (5.217)
PFOA	-	105 (8.15)	102 (9.20)	93.4 (8.04)	96.0 (9.55)	94.0 (9.17)
PFNA	-	104 (9.44)	105 (8.42)	104 (8.33)	102 (9.58)	96.6 (9.69)
PFDA	107 (4.85)	96.2 (6.76)	93.2 (9.95)	91.8 (7.02)	97.4 (8.89)	88.6 (7.49)
PFUnA	-	98.4 (9.41)	91.2 (7.66)	102 (9.44)	100 (10.1)	100 (8.96)
PFDoA	-	109 (5.39)	101 (9.69)	108 (3.90)	109 (6.28)	111 (5.41)
PFTrDA	93.2 (9.53)	105 (8.03)	101 (9.83)	103 (8.29)	110 (8.85)	103 (5.66)
PFTeDA	115 (5.91)	113 (6.22)	114 (3.43)	109 (6.73)	113 (5.98)	113 (2.38)
TFMS	-	-	104 (6.51)	110 (4.55)	108 (7.52)	110 (8.31)
PFEtS	112 (8.35)	100 (10.0)	102 (7.37)	98.4 (9.90)	103 (8.86)	100 (10.3)
PFPrS	101 (8.73)	99.1 (7.37)	103 (5.91)	101 (3.42)	105 (3.07)	108 (4.04)
PFBS	110 (9.58)	100 (9.93)	99.2 (9.05)	102 (7.77)	111 (7.82)	103 (2.68)
PFPeS	-	-	104 (6.34)	100 (5.59)	107 (7.03)	102 (3.90)
PFHxS	105 (9.74)	97.1 (5.50)	97.2 (5.88)	97.9 (8.12)	106 (6.39)	102 (5.89)
PFHpS	103 (8.93)	93.1 (9.22)	98.3 (8.71)	91.0 (6.18)	103 (5.98)	86.7 (8.88)
PFOS	-	108 (8.98)	100 (9.15)	98.3 (5.19)	108 (5.13)	109 (5.53)
PFNS	-	118 (7.54)	99.8 (8.86)	101 (8.79)	115 (6.06)	110 (5.99)
PFDS	-	110 (8.97)	100 (8.63)	105 (9.99)	107 (7.70)	103 (7.84)
PFUdS	109 (11.8)	108 (9.72)	101 (9.71)	105 (7.29)	106 (6.34)	105 (4.41)
PFDoS	-	114 (8.33)	104 (6.72)	106 (7.27)	109 (7.51)	111 (4.69)
PFTrDS	106 (9.37)	111 (4.99)	108 (9.24)	101 (7.75)	108 (7.26)	109 (6.00)
4:2 FTS	95.0 (7.72)	94.6 (10.0)	90.7 (5.21)	94.6 (9.67)	109 (7.20)	94.8 (5.73)
6:2 FTS	97.0 (6.81)	93.8 (9.89)	93.6 (10.6)	88.6 (9.93)	105 (8.29)	89.9 (8.03)
8:2 FTS	94.7 (7.66)	110 (5.56)	102 (8.86)	106 (7.60)	112 (4.14)	102 (9.42)
FOSA	-	-	86.4 (5.30)	100 (8.73)	106 (8.86)	109 (9.15)
NMeFOSA	-	-	-	101 (5.95)	107 (9.34)	88.8 (8.29)
NEtFOSA	-	-	-	99.1 (7.83)	109 (2.97)	90.8 (6.63)
5:3 FTCA	-	-	-	100 (7.89)	95.9 (7.54)	81.3 (4.23)
7:3 FTCA	-	-	-	94.8 (10.6)	89.4 (9.19)	86.9 (6.49)
NMeFOSAA	-	-	-	-	76.4 (8.76)	71.1 (4.74)
NEtFOSAA	-	-	-	-	75.0 (8.01)	74.1 (11.0)
PFMPA	108 (7.29)	98.8 (9.85)	94.5 (7.70)	92.2 (7.52)	100 (6.58)	85.4 (3.52)
PFMBA	106 (9.72)	102 (9.42)	104 (9.19)	105 (8.71)	111 (5.86)	111 (6.33)
HFPO-DA	-	-	-	102 (9.32)	111 (8.12)	111 (6.47)
ADONA	107 (9.56)	104 (9.56)	98.6 (8.03)	102 (9.91)	111 (4.21)	113 (7.52)
PFEESA	101 (9.34)	100 (7.72)	105 (8.36)	105 (5.85)	112 (5.12)	106 (4.67)
9Cl-PF3ONS	111 (9.82)	106 (9.47)	94.7 (7.67)	91.3 (8.82)	96.9 (5.88)	95.9 (6.71)
11Cl-PF3OUdS	111 (5.78)	112 (5.65)	86.4 (5.54)	101 (9.05)	100 (6.18)	98.7 (5.68)

Apple Juice:

Analytes	Average Recovery (RSD, %), n=9					
	Apple Juice					
	Fortified Concentration (ng/L)					
	2	4	10	20	100	500
$^{13}C_2$-TFA	-	-	-	100 (8.34)	95.6 (7.39)	116 (4.89)
PFPrA	-	103 (5.33)	109 (6.40)	104 (5.05)	107 (4.58)	115 (4.46)
PFBA	-	100 (9.92)	105 (9.86)	101 (9.06)	101 (5.41)	112 (8.78)
PFPeA	-	103 (7.73)	105 (7.77)	110 (6.49)	98.0 (4.20)	108 (5.33)
PFHxA	105 (8.21)	100 (8.48)	107 (9.32)	105 (5.56)	101 (8.88)	111 (9.21)
PFHpA	103 (8.42)	104 (8.64)	109 (7.15)	108 (8.42)	108 (3.76)	112 (8.68)
PFOA	105 (10.5)	104 (7.28)	111 (8.64)	113 (7.04)	102 (7.24)	110 (9.30)
PFNA	99.5 (8.21)	103 (9.73)	107 (7.87)	108 (5.42)	98.2 (9.41)	100 (6.70)
PFDA	100 (9.26)	101 (6.55)	106 (9.75)	111 (3.84)	92.8 (8.63)	102 (4.88)
PFUnA	105 (9.15)	107 (9.54)	110 (9.40)	114 (4.80)	98.6 (4.51)	105 (5.11)
PFDoA	100 (9.71)	109 (6.91)	114 (4.47)	114 (6.60)	103 (6.14)	110 (2.07)
PFTrDA	105 (4.88)	109 (3.75)	109 (7.75)	111 (4.93)	106 (5.96)	108 (8.77)
PFTeDA	106 (7.72)	109 (5.79)	108 (7.63)	114 (5.80)	109 (6.67)	110 (7.47)
TFMS	-	-	110 (3.57)	110 (6.49)	107 (3.28)	112 (4.25)
PFEtS	111 (6.55)	104 (9.68)	112 (7.92)	113 (4.56)	96.2 (9.75)	105 (7.20)
PFPrS	102 (9.57)	113 (4.99)	113 (5.94)	113 (6.68)	110 (7.61)	112 (6.55)
PFBS	111 (7.77)	103 (9.12)	111 (8.36)	113 (5.16)	102 (9.78)	108 (4.99)
PFPeS	107 (7.80)	109 (6.29)	115 (6.03)	114 (4.66)	101 (8.29)	111 (4.08)
PFHxS	115 (5.37)	100 (9.99)	107 (9.81)	105 (7.65)	100 (6.73)	109 (2.40)
PFHpS	103 (8.96)	103 (8.83)	110 (7.81)	114 (4.11)	103 (8.55)	107 (8.21)
PFOS	100 (9.28)	105 (7.20)	108 (7.07)	110 (7.20)	109 (7.24)	112 (6.52)
PFNS	94.3 (9.44)	95.7 (8.01)	108 (8.01)	108 (5.01)	103 (8.24)	106 (3.87)
PFDS	114 (9.37)	108 (7.91)	106 (10.1)	114 (3.49)	100 (5.96)	108 (4.84)
PFUdS	108 (9.03)	106 (9.85)	106 (8.80)	112 (4.96)	103 (7.50)	111 (5.14)
PFDoS	117 (7.01)	112 (4.44)	111 (7.11)	110 (7.82)	106 (7.68)	110 (8.99)
PFTrDS	98.1 (7.20)	104 (10.2)	102 (7.95)	107 (5.62)	106 (4.43)	106 (4.68)
4:2 FTS	113 (2.78)	107 (8.03)	113 (4.82)	111 (4.07)	106 (7.23)	108 (7.46)
6:2 FTS	-	103 (5.75)	108 (8.96)	106 (6.80)	113 (2.87)	111 (3.83)
8:2 FTS	98.1 (9.26)	109 (5.66)	108 (8.65)	112 (2.95)	103 (6.38)	110 (8.67)
FOSA	-	113 (7.22)	93.1 (8.26)	108 (6.88)	101 (8.20)	112 (4.83)
NMeFOSA	-	-	98.9 (8.53)	111 (7.32)	100 (10.8)	104 (7.25)
NEtFOSA	-	-	102 (7.18)	109 (6.10)	83.6 (3.59)	98.5 (6.59)
5:3 FTCA	-	-	-	104 (8.88)	104 (8.63)	107 (9.66)
7:3 FTCA	-	-	-	107 (8.80)	97.8 (9.49)	91.5 (9.88)
NMeFOSAA	-	-	94.9 (9.09)	109 (9.19)	105 (9.36)	112 (4.56)
NEtFOSAA	-	-	108 (9.13)	106 (8.94)	93.7 (10.6)	111 (5.95)
PFMPA	-	92.4 (8.56)	98.4 (9.49)	103 (7.70)	95.5 (4.59)	105 (8.86)
PFMBA	97.6 (5.16)	102 (5.63)	109 (9.29)	110 (8.37)	94.5 (7.43)	104 (5.55)
HFPO-DA	-	-	117 (3.35)	112 (4.40)	110 (7.84)	113 (3.93)
ADONA	93.9 (7.70)	98.7 (9.16)	106 (8.27)	106 (9.42)	97.2 (4.43)	103 (9.28)
PFEESA	109 (7.55)	104 (4.63)	110 (8.58)	108 (6.43)	95.9 (9.48)	104 (3.72)
9Cl-PF3ONS	102 (7.89)	110 (10.0)	112 (6.40)	110 (6.53)	94.9 (7.97)	100 (3.36)
11Cl-PF3OUdS	109 (5.77)	108 (4.73)	110 (7.77)	109 (6.35)	99.0 (5.55)	105 (3.19)

Blended Berry Juice:

Analytes	Average Recovery (RSD, %), n=9 Blended Berry Juice Fortified Concentration (ng/L)					
	2	4	10	20	100	500
$^{13}C_2$-TFA	-	-	-	94.3 (5.65)	104 (1.94)	114 (0.88)
PFPrA	-	110 (9.34)	110 (5.05)	107 (7.17)	111 (4.92)	105 (3.80)
PFBA	-	109 (8.06)	110 (8.65)	108 (6.62)	104 (9.48)	94.1 (6.35)
PFPeA	-	102 (8.13)	106 (8.92)	94.8 (9.61)	105 (4.38)	101 (8.78)
PFHxA	106 (8.34)	99.1 (9.69)	97.6 (9.07)	95.6 (5.25)	104 (2.97)	99.6 (7.18)
PFHpA	106 (9.74)	104 (8.49)	110 (7.86)	107 (9.03)	107 (8.88)	103 (7.69)
PFOA	106 (6.78)	107 (8.53)	105 (7.54)	108 (5.82)	102 (5.41)	101 (9.17)
PFNA	106 (9.47)	106 (6.45)	103 (4.80)	108 (6.15)	97.2 (8.85)	96.3 (5.25)
PFDA	100 (8.41)	109 (8.62)	112 (4.56)	114 (4.42)	104 (9.84)	108 (5.66)
PFUnA	104 (8.55)	106 (5.85)	103 (8.94)	110 (5.94)	96.5 (6.33)	101 (7.31)
PFDoA	111 (6.98)	109 (7.06)	108 (6.30)	115 (3.95)	97.9 (6.15)	104 (7.28)
PFTrDA	102 (4.85)	108 (6.37)	97.2 (9.78)	111 (6.43)	99.6 (6.35)	108 (9.52)
PFTeDA	106 (4.73)	105 (4.56)	96.6 (7.63)	114 (3.89)	102 (3.29)	109 (8.41)
TFMS	-	-	105 (4.65)	109 (3.53)	111 (5.55)	114 (4.54)
PFEtS	113 (5.75)	109 (6.75)	111 (6.78)	112 (6.59)	109 (7.19)	112 (4.10)
PFPrS	104 (9.93)	105 (7.84)	110 (8.21)	106 (6.13)	110 (4.55)	110 (9.02)
PFBS	110 (9.59)	101 (9.58)	106 (5.83)	98.5 (6.93)	108 (5.87)	96.8 (2.27)
PFPeS	102 (8.01)	103 (8.47)	100 (4.04)	104 (7.94)	111 (8.67)	100 (4.41)
PFHxS	121 (2.63)	97.9 (7.91)	102 (7.27)	97.3 (7.45)	101 (5.40)	98.7 (7.35)
PFHpS	104 (9.71)	107 (7.04)	110 (6.22)	112 (4.28)	111 (4.77)	99.3 (5.76)
PFOS	109 (8.60)	104 (9.08)	99.4 (9.43)	107 (4.43)	105 (6.60)	109 (9.20)
PFNS	106 (10.0)	106 (9.05)	103 (9.53)	113 (3.87)	99.4 (5.39)	102 (8.75)
PFDS	117 (5.26)	115 (7.64)	104 (10.1)	110 (6.35)	99.2 (7.59)	106 (8.96)
PFUdS	110 (9.55)	99.4 (9.91)	102 (8.31)	114 (3.77)	97.7 (5.54)	105 (9.64)
PFDoS	112 (8.38)	114 (7.34)	106 (7.36)	111 (6.22)	98.5 (4.55)	109 (7.70)
PFTrDS	118 (5.45)	105 (8.22)	107 (9.63)	111 (7.64)	98.6 (5.14)	109 (9.31)
4:2 FTS	113 (4.84)	96.7 (9.18)	100 (9.62)	93.7 (9.99)	92.5 (9.98)	95.4 (9.41)
6:2 FTS	-	97.9 (9.49)	103 (9.92)	95.8 (5.54)	95.3 (7.85)	101 (9.59)
8:2 FTS	97.9 (8.92)	94.9 (9.01)	108 (4.02)	99.4 (7.84)	92.5 (6.18)	93.1 (5.47)
FOSA	-	112 (9.69)	95.8 (10.3)	108 (9.65)	100 (9.18)	96.5 (7.23)
NMeFOSA	-	-	107 (6.32)	108 (9.18)	105 (9.20)	107 (5.20)
NEtFOSA	-	-	97.5 (9.70)	114 (4.09)	99.2 (9.06)	104 (8.33)
5:3 FTCA	-	-	-	107 (9.06)	110 (5.34)	101 (6.95)
7:3 FTCA	-	-	-	103 (8.73)	100 (6.43)	87.6 (9.60)
NMeFOSAA	-	111 (5.76)	106 (3.40)	102 (3.10)	78.5 (7.91)	111 (2.71)
NEtFOSAA	-	-	102 (9.91)	108 (8.12)	95.2 (9.76)	94.2 (9.68)
PFMPA	103 (9.19)	98.1 (9.01)	98.2 (8.02)	90.9 (8.82)	100 (9.17)	91.6 (4.13)
PFMBA	106 (3.93)	95.0 (6.39)	106 (9.20)	92.8 (8.93)	104 (3.69)	96.8 (4.67)
HFPO-DA	-	-	-	108 (9.00)	99.1 (10.3)	113 (4.02)
ADONA	111 (9.21)	103 (9.02)	95.7 (9.26)	92.8 (8.61)	99.0 (7.31)	92.0 (4.84)
PFEESA	101 (9.28)	95.8 (9.76)	101 (6.99)	100 (7.70)	107 (3.21)	95.1 (3.23)
9Cl-PF3ONS	102 (8.98)	101 (9.11)	97.8 (7.91)	109 (5.06)	99.3 (4.23)	101 (7.61)
11Cl-PF3OUdS	109 (6.95)	117 (3.67)	109 (6.38)	115 (5.11)	97.3 (2.80)	103 (7.69)

Orange Juice:

Analytes	Average Recovery (RSD, %), n=9					
	Orange Juice					
	Fortified Concentration (ng/L)					
	**2**	**4**	**10**	**20**	**100**	**500**
$^{13}C_2$-TFA	-	-	-	109 (4.78)	97.0 (7.06)	114 (3.48)
PFPrA	-	-	112 (9.01)	101 (8.91)	111 (4.88)	113 (2.79)
PFBA	-	104 (6.26)	102 (6.19)	106 (5.90)	109 (6.11)	105 (2.32)
PFPeA	-	112 (8.29)	107 (8.34)	97.6 (8.75)	103 (5.43)	104 (3.63)
PFHxA	-	108 (8.53)	102 (7.96)	98.7 (9.41)	106 (7.22)	102 (5.65)
PFHpA	-	107 (9.56)	104 (8.25)	109 (4.34)	113 (9.31)	114 (2.85)
PFOA	-	112 (5.83)	101 (9.36)	93.8 (8.96)	99.2 (7.07)	90.8 (3.00)
PFNA	-	107 (7.92)	97.1 (9.17)	95.0 (7.20)	99.3 (8.94)	95.3 (4.97)
PFDA	-	105 (9.58)	97.1 (8.98)	89.6 (7.06)	103 (4.58)	95.3 (5.14)
PFUnA	-	103 (8.83)	102 (7.80)	98.7 (9.25)	104 (7.57)	92.3 (2.80)
PFDoA	-	118 (4.35)	104 (9.54)	103 (9.09)	111 (5.50)	97.7 (2.60)
PFTrDA	-	111 (4.93)	101 (8.79)	95.7 (8.87)	96.6 (4.50)	86.5 (2.11)
PFTeDA	-	115 (6.24)	106 (5.81)	101 (6.93)	111 (6.56)	104 (1.60)
TFMS	-	-	-	105 (7.35)	108 (5.60)	102 (3.51)
PFEtS	-	110 (6.57)	108 (5.56)	109 (5.22)	108 (2.56)	112 (4.06)
PFPrS	-	109 (5.62)	110 (6.21)	105 (9.07)	107 (7.16)	109 (5.84)
PFBS	-	107 (9.79)	102 (7.49)	99.6 (9.76)	105 (6.19)	102 (3.54)
PFPeS	-	106 (9.30)	105 (7.37)	108 (8.49)	107 (6.11)	112 (7.53)
PFHxS	-	111 (7.18)	99.0 (7.59)	96.0 (6.94)	95.9 (9.08)	101 (6.24)
PFHpS	-	111 (5.92)	98.3 (9.71)	102 (8.34)	100 (6.60)	102 (2.52)
PFOS	-	114 (8.86)	103 (9.19)	94.3 (8.99)	96.8 (6.13)	98.9 (4.38)
PFNS	-	120 (7.66)	86.8 (5.72)	102 (10.1)	95.2 (8.09)	92.4 (3.12)
PFDS	-	111 (7.72)	97.2 (8.59)	95.7 (10.7)	95.1 (6.71)	83.9 (3.69)
PFUdS	-	112 (6.21)	99.7 (8.65)	96.1 (9.44)	100 (6.93)	90.4 (3.81)
PFDoS	-	109 (6.06)	98.3 (9.53)	98.8 (9.43)	106 (6.57)	93.6 (3.54)
PFTrDS	-	115 (5.44)	109 (6.27)	95.9 (10.6)	104 (7.01)	95.0 (5.06)
4:2 FTS	-	102 (8.22)	104 (8.92)	93.0 (6.36)	98.4 (9.32)	93.4 (6.29)
6:2 FTS	-	104 (3.08)	97.7 (7.27)	94.4 (9.75)	110 (7.26)	109 (5.80)
8:2 FTS	-	108 (8.43)	96.5 (8.58)	103 (8.41)	108 (8.02)	115 (3.39)
FOSA	-	116 (8.38)	101 (7.95)	102 (9.79)	101 (8.26)	107 (8.98)
NMeFOSA	-	-	103 (9.25)	107 (9.74)	109 (7.03)	115 (3.60)
NEtFOSA	-	-	107 (4.13)	106 (7.54)	104 (9.22)	100 (9.16)
5:3 FTCA	-	-	-	94.5 (9.06)	100 (8.37)	100 (6.81)
7:3 FTCA	-	-	-	99.3 (9.08)	109 (6.13)	111 (3.62)
NMeFOSAA	-	-	122 (2.96)	99.4 (8.85)	106 (8.90)	109 (6.56)
NEtFOSAA	-	-	-	94.7 (9.55)	102 (9.15)	102 (9.44)
PFMPA	-	100 (7.26)	95.7 (7.44)	93.5 (7.34)	93.1 (7.00)	87.3 (4.14)
PFMBA	-	111 (4.98)	107 (8.50)	91.7 (6.34)	110 (6.47)	103 (5.88)
HFPO-DA	-	-	-	91.9 (8.61)	104 (9.74)	88.5 (8.47)
ADONA	-	102 (9.87)	99.4 (8.22)	94.4 (6.41)	109 (5.66)	107 (4.46)
PFEESA	-	110 (6.86)	96.9 (9.85)	91.4 (9.96)	102 (7.64)	98.9 (1.32)
9Cl-PF3ONS	-	111 (3.64)	97.8 (8.46)	104 (9.83)	107 (6.47)	103 (2.06)
11Cl-PF3OUdS	-	108 (5.31)	91.8 (7.72)	93.0 (7.28)	98.4 (3.61)	87.3 (1.49)

Table S4. Measurement of PFAS in commercial beverage samples

Beverage Samples	Concentration (ng/L)											
	Ultrashort-Chain				Short-Chain			Long-Chain				Alternatives
	TFA	TFMS	PFPrA	PFPrS	PFBA	PFBS	PFPeS	PFOA	PFOS	PFNA	PFTrDA	PFEESA
Tea												
Green Tea, unsweetened	4,224	24.6	57.8	2.95	31.9	< 2.00	nd*	nd	nd	nd	nd	nd
Green Tea, sweetened	1,243	2.11	4.79	4.08	5.71	< 2.00	< 2.00	nd	nd	nd	nd	nd
Black Tea, unsweetened #1	1,250	16.3	nd	38.4	nd	nd	24.4	nd	nd	nd	4.67	nd
Black Tea, unsweetened #2	827	nd	nd	96.9	nd	nd	18.7	nd	nd	nd	< 2.00	nd
Black Tea, sweetened #1	644	3.00	nd	15.4	nd	nd	35.2	nd	nd	nd	nd	nd
Black Tea, sweetened #2	545	< 2.00	nd	38.6	nd	nd	11.1	nd	nd	2.16	3.41	2.32
Lemon Black Tea	928	14.4	nd	31.3	nd	nd	26.4	nd	nd	nd	3.22	nd
Raspberry Black Tea	859	3.37	nd	23.1	nd	nd	34.4	nd	nd	nd	6.71	nd
Juice												
Apple Juice #1	761	4.84	3.69	nd	3.77	nd	nd	< 2.00	nd	nd	< 2.00	< 2.00
Apple Juice #2	1,638	21.7	204	nd	45.8	nd	nd	nd	nd	nd	nd	nd
Blended Berry Juice #1	1,030	2.87	nd	nd	nd	nd	nd	< 2.00	nd	nd	< 2.00	nd
Blended Berry Juice #2	2,732	65.1	35.9	nd	nd	4.39	nd	3.78	8.40	nd	nd	nd
Cranberry Juice #1	2,185	16.6	25.5	nd	nd	nd	nd	nd	nd	nd	nd	nd
Cranberry Juice #2	12,372	5.81	153	nd	37.4	nd	nd	nd	nd	nd	nd	nd
Cranberry-Raspberry-Lemonade Juice	2,103	11.6	17.2	nd	nd	nd	nd	nd	nd	nd	nd	nd
Cranberry-Apple-Raspberry Juice	836	5.50	nd	nd	nd	nd	nd	nd	nd	nd	nd	nd
Grape Juice	27,185	43.7	184	96.2	159	nd	nd	nd	nd	nd	nd	nd
Orange Juice #1	35,188	76.9	nd	nd	12.9	nd	nd	nd	nd	nd	nd	nd
Orange Juice #2	18,361	224	nd	nd	nd	nd	nd	nd	nd	nd	nd	nd
Orange Juice #3	19,605	112	nd	nd	45.4	nd	nd	nd	nd	nd	nd	nd
Orange Peach Mango juice	6,486	205	24.3	nd	nd	nd	nd	nd	nd	nd	nd	nd
Pineapple Juice	12,228	28.0	35.5	nd	nd	nd	nd	nd	nd	nd	nd	nd
Strawberry Kiwi	869	7.05	6.40	nd	nd	nd	nd	nd	nd	nd	nd	nd

*non-detected
